# Patient empowerment in risk management: a mixed-method study to explore mental health professionals’ perspective

**DOI:** 10.1186/s12913-019-4215-x

**Published:** 2019-06-13

**Authors:** M. Rimondini, I. M. Busch, M. A. Mazzi, V. Donisi, A. Poli, E. Bovolenta, F. Moretti

**Affiliations:** 1 Section of Clinical Psychology, Department of Biomedicine, Neuroscience and Movement, University of Verona, Verona, Italy; 2 Section of Hygiene and Preventive Medicine, Department of Diagnostic and Public Health, University of Verona, Verona, Italy

**Keywords:** Patient-safety, Risk management, Risk assessment, Psychiatry, Mental health, Patient empowerment, Focus group, Mixed-method

## Abstract

**Background:**

In the last years, patients’ empowerment has been increasingly recognized as a crucial dimension of patient-centered healthcare and patient safety. Nevertheless, little work has been done so far in the field of patient safety to investigate strategies for empowering psychiatric patients. Therefore, the aim of this study was to identify, by using focus groups, whether and how psychiatric patients’ empowerment can improve risk management according to the perspective of healthcare providers (HPs).

**Methods:**

A mixed-method approach composed of a qualitative data collection method (i.e., focus groups) and a quantitative analysis technique (i.e., inductive content analysis) was applied. HPs working in mental health settings shared their perspectives on psychiatric patients’ empowerment in risk management. After the transcription of the audio-taped discussions and the subsequent development of a hierarchical four-level coding system (strategy versus critical issue, thematic area, category, subcategory), two independent raters codified the transcripts and synthesized the content. Absolute frequencies are reported for quantitative data.

**Results:**

Twelve focus groups consisting of six to ten participants, each with an overall sample size of 95 participants (65 women; average age ± SD 47 ± 9 yrs), were enrolled. A total of 1252 participants’ verbal contributions (i.e., units of analysis) were assessed. Strategies and critical issues (Level 1) were mentioned almost equally (52 and 48%, respectively) by the HPs. Most of the contributions at Level 2 referred to the thematic areas *Treatment and Cure* (69%) and *Emergency Management* (21%). In the area *Treatment and Cure*, the category *Therapeutic Compliance* (Level 3) was discussed in one third of all contributions.

**Conclusions:**

Our results suggest that HPs consider patients as crucial partners in risk management and expect them to play a key role in actively enhancing safety. Policy makers should be aware that risk management in mental health settings particularly relies on the therapeutic relationship between HPs and patients. Therefore, allocating sufficient human and financial resources to mental health care aiming to further support the relationship between patients and HPs is of utmost importance.

**Electronic supplementary material:**

The online version of this article (10.1186/s12913-019-4215-x) contains supplementary material, which is available to authorized users.

## Background

In the last years, patients’ empowerment has been increasingly recognized as a crucial dimension of patient-centered healthcare [1] that can improve patients’ understanding and control over their health and healthcare process [2]. Instead of only passively receiving healthcare assistance, patients can become, to some extent, self-determining and independent [3, 4] with a potential positive impact on several health outcomes such as treatment adherence and self-care practices [5, 6]. Furthermore, improving the quality of care through the involvement of patients may lead to positive outcomes also in terms of patient safety. Already in 2004, the World Health Organization (WHO) [7] declared patients’ active role and involvement in their own path of care as essential for enhancing patient safety; the same approach, aiming to guarantee a better patients’ safety, was postulated in 2009 also by the European Council [8].

Despite the increasing acknowledgement of the importance of patients’ role in healthcare, patients’ perspective in risk management is still underestimated [9]. Recently, intending to overcome this misestimation, Peat et al. [10] proposed a framework to conceptualize possible routes by which patients may be involved in their care and give a substantial contribution to improve their own safety. For instance, patients can help to assure that an appropriate treatment is correctly administered by sharing significant health information with healthcare providers and by asking questions regarding the chosen type of treatment.

The necessity to further promote patient empowerment in risk management is also underlined by the key role that the human factor may play in ensuring safety [11]. Indeed, according to the traditional risk management approach, *human fallibility* exists and can be efficiently addressed by introducing changes in the system and improving defenses which can avert adverse events and mitigate their effects [12]. Such an approach assumes that human variability represents a potential threat to patient safety and needs to be reduced by appropriate systemic changes. On the contrary, the most recent literature proposed a different view of *human variability*, addressing it as a precious resource strengthening system flexibility and resilience (i.e., the capability of a system to handle unexpected, unsafe variations by rapidly restoring an acceptable level of variability able to prevent the occurrence of adverse events) [11].

According to this evidence, not only healthcare providers but also patients and patients’ caregivers, representing the sharp end of any healthcare process, are supposed to play a key role in actively carrying out tasks and enhancing safety [11]. For instance, Weingart and colleagues [13] demonstrated that patients were able to identify medical errors (i.e., potential source of system variations) not recognized by their physicians or any other healthcare provider involved in their care. Despite these recognized advantages of patients’ involvement in risk management, also potential intrinsic challenges have to be fully considered and faced. For instance, the fear of healthcare providers of a power shift to patients [14] may impede the implementation of patient empowerment. Considering the current focus on performance accountability [15] and the insurmountable expectation of perfection [11, 16] affecting healthcare providers, the “empowered” patient, who actively participates in her/his own safety may then be seen not as a partner but rather as a threatening figure pointing out healthcare providers’ wrongdoing or even taking legal action [17]. At the same time, it also has to be considered, as highlighted by Doherty and Stavropoulou [18], that patients perceive themselves as vulnerable as well as dependent on their healthcare providers. For some patients, the feeling of being co-responsible for safe treatment delivery may lead to increased anxiety and may represent a burden [10]. These barriers might be particularly challenging in the mental healthcare setting in which the proper balance between a safe level of control and an approach taking into account patients’ perspective is difficult to achieve.

While many principles of patient safety – mostly derived from studies performed in medical health care settings such as acute and emergency care – have been directly transferred to mental health settings, several patient safety aspects, unique to mental health care (e.g., self-harm, suicide, restraint use), still have to be fully addressed [19–22], especially considering that adverse events are common [23, 24] and carry a high socio-economic impact [25].

Additionally, to the necessity to further investigate the unique challenges of safety in mental health care, the need to increase the active involvement of patients suffering from psychiatric disease have been wildly acknowledged as a priority [2, 26, 27]. Nevertheless, little work has been done to investigate strategies to empower mentally ill patients in the field of patient safety considering healthcare providers’ [28] and patients’ perspectives.

Therefore, the main aim of this study is, by using focus groups, to identify whether and how psychiatric patients’ empowerment can improve risk management according to the perspective of healthcare providers (HPs). In particular, our study aims to investigate the critical issues and strategies related to psychiatric patients’ empowerment in risk management, since, as above-mentioned, this group of patients is especially vulnerable.

## Methods

### Recruitment and focus groups

Ethical approval for the present study was obtained by the Ethical Committee of the Verona University Hospital (Protocol n.16160; 31/03/2016).

The study was conducted within the Verona Mental Health Department (MHD) in the Northeast of Italy (459,536 inhabitants). Specifically, four mental health services and one “specialized penitentiary mental healthcare unit” (i.e., a small-scale facility for prisoners with mental health problems that, according to Italian law, has recently replaced forensic hospitals) were included. The MHD includes: 1) four 15-bed acute inpatient wards (i.e., open wards with dedicated staff where patients can be admitted on a voluntary or compulsory basis) located in three general hospitals; 2) four community mental health centers providing day care and rehabilitation; 3) 13 outpatient clinics, providing emergency care and continuity of care as well as scheduled domiciliary visits; 4) one liaison service offering psychiatric and psychological consultations for other departments of the general hospital, 5) one 24-h emergency department, and 5) 36 sheltered accommodations. We recruited participants from various MHD settings to encourage different perceptions and points of view and to thus increase representativeness and generalizability of our findings. All the HPs of the MHD having attended a continuing educational course on risk management in 2016 were invited to participate in the study. Indeed, we considered such a common background in risk management as a useful prerequisite for guiding participants more easily during the focus group discussions. All recruited participants signed the informed consent form and were then divided into 12 focus groups of six to 10 participants.

Experienced focus group facilitators introduced the rules highlighting the importance of confidentiality. Participants were then encouraged to provide their opinions derived from their professional experiences and knowledge in the field of mental health and patient safety. According to the aims of this study, the following questions were proposed one by one to stimulate the discussion:

1. What do you think about patients’ involvement in risk management in psychiatry?

2. Which are the potential limits/risks of involving psychiatric patients in risk management?

3. Which are the potential benefits/strengths of involving psychiatric patients in risk management?

4. Which strategies do you usually apply in your daily practice in order to engage patients in their safety?

In the subsequent one-hour discussion, participants exchanged their opinions but were asked to adhere as much as possible to the themes proposed by the facilitators and to respect turn-taking. Facilitators had also to promote the debate and ensure the active involvement of each participant without interfering with the content of the discussion.

### Inductive content analysis and development of the four-level hierarchical coding system

We applied a mixed-method approach, since it is one of the most effective methodologies able to “capture the complexity of healthcare processes and to gather advanced insight into healthcare communication phenomena” (p.281) [29]. By combining quantitative and qualitative techniques, a large amount of information, collected in flexible and iterative ways, can be synthetized and analyzed.

The rationale for using multiple forms of research is based on the understanding that all methods have strengths and limitations and that the advantages of quantitative research can outweigh the disadvantages of qualitative research and vice versa.

**Fig. 1:**
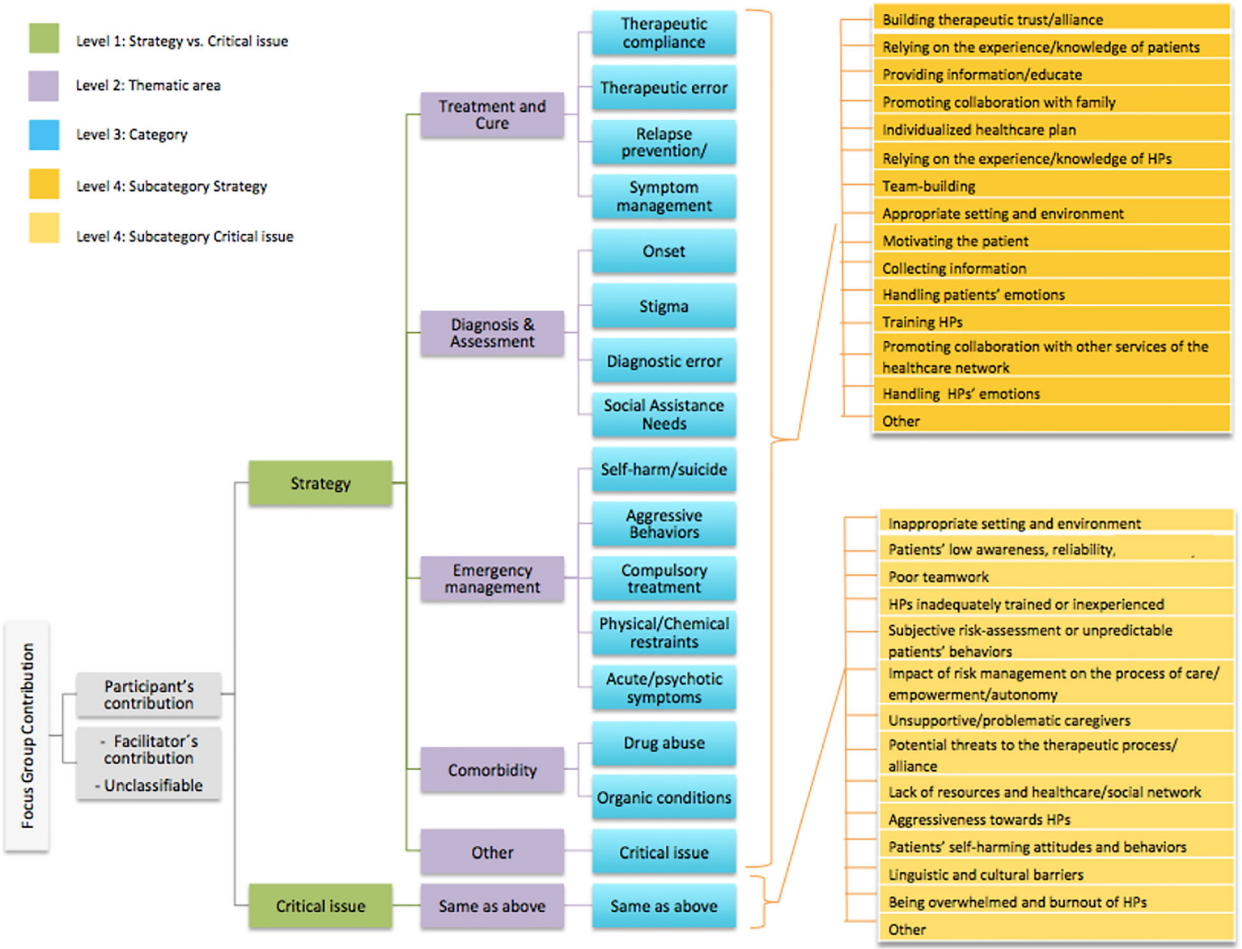
Hierarchical structure of the coding system developed in order to synthesize the focus group content

All focus group discussions, including facilitators’ questions and comments, were audiotaped and fully transcribed. In order to synthesize the transcriptions, two researchers (MR and FM) independently analyzed the discussion of one randomly selected focus group following the guidelines for qualitative content analysis [30]. Each researcher preliminary labeled all verbal contributions (i.e., phrases, comments, opinions, suggestions of each participating HP) according to the above-mentioned questions and then sub-grouped them in areas conveying similar concepts. After this first round of individual text analysis, the researchers discussed and merged their preliminary labels into a unique coding system, organized in a hierarchical structure of four levels (see Fig. 1).

In order to verify if all topics raised by the participants could be captured by the coding system, two researchers separately coded the transcripts of two additional focus group discussions. Since no additional topics emerged, the coding system was considered exhaustive (i.e., saturation criterion) and the four levels were confirmed as follows:

Level 1 aims to distinguish between HPs’ contributions describing or referring to a strategy (i.e., a statement underlying a way patient empowerment may improve risk management) and HPs’ contributions pointing out a critical issue (i.e., a statement underlying critical aspects that may hinder patient empowerment in risk management). Level 2 (*thematic areas)* refers to different aspects of the process of care (i.e., *Treatment and cure*, *Diagnosis and assessmen*t, *Emergency management*, *Comorbidity*, *Other*). Level 3 (*Categories)* consists of specific issues related to certain thematic areas (e.g., the thematic area *Comorbidity* was divided into the two categories *Drug abuse* and *Organic Conditions*). Finally, level 4 lists specific strategies or critical issues applicable to each category at level 3 (e.g., the strategy *Handling HPs emotions* might be referred to several categories, such as *Therapeutic compliance*, *Diagnostic error*, or *Compulsory treatment*).

In order to ensure that facilitators’ interventions did not influence the course of participants’ discussion, one researcher (MR) verified facilitators’ neutrality by analyzing all their verbal turns in the transcripts. To provide definitions and rules for coding each transcript of each focus group, a manual was developed (MR and FM) (see additional file 1 for the definitions of thematic areas and categories).

Two independent raters (VD and EB) then coded all the contributions of all focus groups according to the established four-level hierarchical coding system and by using the above-mentioned manual.

Aiming to evaluate the reliability of the coding system, the inter-rater agreement was evaluated for one randomly selected focus group transcript. The inter-rater agreement turned out to be good at Level 1 (87%, κ = 0.73, 95% CI [0.607, 0.862]), moderate at level 2 (72%, κ = 0.55, 95% CI [0.420, 0.686]) and at level 3 (64%, κ = 0.55, 95% CI [0.425, 0.673]). The fourth and last level was characterized by skewed data and empty cells, inter-rater reliability analysis could therefore not be performed. The overall inter-rater agreement was considered satisfying. In order to further improve the quality of the coding process, in particular for level 4, it was established that unsure codings had to be discussed and resolved in consensus.

To further assess the robustness of the coding system, 1 year after data collection (i.e., 2017), all study participants were invited to a meeting. A subsample of the study participants (*n* = 50) attended this encounter and discussed the coding system, the thematic areas and a synthesis of the study results. The majority of participants’ comments was positive, and no additional areas of interest were suggested, demonstrating the comprehensiveness of the applied coding system.

### Statistical analysis

Interrater reliability of the coding system has been performed with Cohen’s Kappa by using STATA module Kappaetc [31].

Following a mixed-method approach [30], a frequency distribution of each thematic area, category, and subcategory is provided together with fragments of text extracted from the focus group transcripts. In order to check if all participants contributed to the focus groups discussions, we ensured to maintain the link between each contributing statement and its author in a sub-sample of four focus-groups. Rates of occurrence of all the expressions by participant were calculated.

## Results

Twelve focus groups with an overall sample size of 95 participants (65 women, 30 men; average age ± SD 47 ± 9 yrs) were enrolled. Participants were psychiatric nurses (*n* = 67), healthcare and social assistance operators (*n* = 10), and other mental health professionals (e.g., psychiatrists, clinical psychologists). Most of the participants (59%) had 10 to 30 years of work experience, 19% more than 30 years, and 19% one to 9 years, the remaining (3%) were HPs hired during the last year. Most of the participants (68.4%) reported to have already experienced at least one adverse event in their career.

The total number of HPs’ verbal contributions and facilitators’ interventions was 1252. Seven hundred sixty-three and 348 out of 1252 were HPs’ contributions and facilitators’ interventions, respectively. The analysis of the occurrence rate of each participant’s verbal contributions confirmed that all HPs contributed to the focus groups (range of contributions: 9–59). One hundred forty-one contributions were excluded since they consisted of very short, unclassifiable sentences (e.g., “*I don’t know… actually it depends… it is a difficult topic*”).

Facilitators’ most frequent interventions were open, general questions (e.g., “*anything else? Do you all agree?*”), back channel responses (e.g., “*hmm, please continue…*”), reassurance (e.g., “*there are no right answers for this discussion.*”), transitions (e.g., “*sorry to interrupt the discussion, but this content doesn’t fully fit to the topic of risk management…*”), and synthesis (e.g., “*correct me if I’m wrong, but according to your opinion the main strategies applied during compulsory treatments are…..*”).

Regarding level 1 of the coding system, HPs almost equally addressed strategies (52%) and critical issues (48%) (see Table 1).

**Table 1:** Frequency distribution and percentages of HPs’ contributions coded at Level 1, 2 and 3

Thematic area	Category	Strategy	Critical issue	Total
Treatment and Cure	Therapeutic compliance	142 (35.4%)	114 (31.5%)	256 (33.6%)
	Therapeutic error	50 (12.5%)	28 (7.7%)	78 (10.2%)
	Relapse prevention/symptom management	114 (28.4%)	81 (22.4%)	195 (25.6%)
Diagnosis and Assessment	Onset	0 (0%)	6 (1.7%)	6 (0.8%)
	Stigma	3 (0.7%)	4 (1.1%)	7 (0.9%)
	Diagnostic error	5 (1.2%)	2 (0.6%)	7 (0.9%)
	Social assistance needs	13 (3.2%)	24 (6.6%)	37 (4.8%)
Emergency management	Self-harm/suicide	19 (4.7%)	17 (4.7%)	36 (4.7%)
	Aggressive behaviors	23 (5.7%)	42 (11.6%)	65 (8.5%)
	Compulsory treatment	10 (2.5%)	11 (3%)	21 (2.8%)
	Physical/Chemical restraints	5 (1.2%)	7 (1.9%)	12 (1.6%)
	Acute/Psychotic symptoms	13 (3.2%)	12 (3.3%)	25 (3.3%)
Comorbidity	Drug abuse	0 (0%)	5 (1.4%)	5 (0.7%)
	Organic conditions	1 (0.2%)	3 (0.8%)	4 (0.5%)
Other		3 (0.7%)	6 (1.7%)	9 (1.2%)
Total		401	362	763

At level 2, the most frequently discussed thematic area was *Treatment and cure* (69%) that included all participants’ verbal contributions regarding patients’ empowerment in risk management during the process of care (i.e., referring either to pharmacological treatment or any other therapeutic approach planned for the patient). Participants discussed also the involvement of patients in the safety management during acute phases of the disease (e.g., severe psychotic symptoms) and in emergency settings (e.g., patient receiving compulsory medical treatment) (*Emergency management*, 21%). Finally, strategies and critical issues regarding patients’ role in risk reduction related to the diagnosis and assessment of mental disorders were only rarely addressed (*Diagnosis and Assessment*, 7%).

At level 3, the role of *Therapeutic compliance* in ensuring patient safety (i.e., regarding both compliance to pharmacological therapy and to any other aspect of the management of disability) was discussed in one third (34%) of all the HPs’ contributions. Other categories frequently mentioned as relevant in risk management were: *Relapse prevention/symptom management* (26%*)*, *Therapeutic error* (i.e., errors occurring in the administration and management of the pharmacological therapy, 10%), and *Aggressive behaviors* toward others (e.g., states of psychomotor agitation, 8%).

Tables 2 and 3 show, respectively, the frequency distribution of strategies and critical issues (subcategories at level 4) independently from the thematic area.

**Table 2:** Frequency distribution and examples of the focus groups contributions included in the *Strategies* subcategory (Level 4)

Strategy	*n*	Examples
Building therapeutic trust/alliance	73	*“Often patients are aggressive because they are scared. In order to face these reactions and to gain patients’ trust and alliance, it is important that all HPs are self-confident and convey reassurance…”*
Relying on the experience/knowledge of patients	58	*“When patients know their pharmacological therapy, they may be able to recognize and report eventual administration errors.”*
Providing information/educate	58	*“We organize patient group meetings in which we discuss about the effects of the drugs and the potential side effects in terms of pros and cons. The aim is to favour the autonomy, these groups work, patients have shown more self-awareness.”*
Promoting collaboration with family	35	*“Team working does not refer only to the collaboration between HPs and patients. It deserves also family involvement because it is important for patients to feel supported by their relatives and, on the other hand, family member can provide important information to clinicians.”*
Individualized healthcare plan	34	*“It’s important to listen to patients without prejudice. Sometimes you might have a project for that patient, which then turns out to be far away from his expectations. During rehabilitation you can’t follow your own direction without taking into account what patients want, can do and would like to do. Otherwise, the risk of failure is very high”*
Relying on the experience/knowledge of HPs	33	*“Human factor in dealing with patients’ aggressive behaviours is crucial, since it is difficult to have a rule that can be applied to all patients. HPs decide to react to patients’ aggression on the basis of the knowledge that they have about that specific person.”*
Team-building	25	*“It is important that the team has a common goal, since it is not the single person that can make a difference…”*
Appropriate setting and environment	18	*“It is important that patients can be welcomed in a comfortable environment, maybe with a garden where they can smoke…”*
Motivating the patient	17	*“Helping patients to understand the importance of respecting the rules may lead to better results instead of simply imposing prohibitions”*
Collecting information	17	*“…in order to involve patients in risk management, it would be a good idea to directly ask them what they expect. Collecting their advices and requests may let us understand needs that we may not expect”*
Handling patients’ emotions	12	*“The risk of aggressions is reduced by encouraging patients to express their emotions. For example, if they are feeling some aggressive drive against other people and they are free to express and discuss it, then this is a first step for reducing internal tension.”*
Training HPs	8	*“Professionals who want to work in psychiatry have to be well trained. It’s inappropriate to select them randomly from internal rankings. If you want to reduce risk in psychiatry, the first step is healthcare providers’ training.”*
Promoting collaboration with other services of the healthcare network	5	*“Before an involuntary treatment, it’s important to inform the police that will be present in order to share information on the patient and on the reasons underlying this intervention”*
Handling HPs’ emotions	5	*“…to recognize and prevent burnout, help HPs to better cope with their feelings, eliminate the elements that might have brought the HP to the physical and mental exhaustion, like excessive workload…”*
Other	3	*“I think that another area in which patients might be involved regarding their safety is the personal hygiene...most of our patients are reluctant to take a shower, but in that moment, you might teach them how to get out from the shower box without falling…”*
Total	401

**Table 3:** Frequency distribution and examples of the focus groups contributions included *in the Critical Issue subcategory* (Level 4)

Critical Issue	n	Examples
Inappropriate setting and environment	45	*“The environment would be adequate for a medical setting rather than a psychiatric setting; a psychiatric patient needs more open spaces to move safely and get relaxed…”*
Patients’ low awareness, reliability	43	*“We can share the responsibility with the patient only when he has totally understood and accepted what is happening to himself, otherwise it is very difficult…”*
Poor teamwork	38	*“Another risk is the lack of communication between different members of the team…the team communicates only regarding the diagnosis… each healthcare professional brings his personal point of view and works with the patient focusing only on his specific field.. there is no space for sharing different perspectives…”*
HPs inadequately trained or inexperienced	37	*“I have never done any training on this topic. I know that I may change my attitude towards the patients, but I don’t know how to do it”*
Subjective risk-assessment or unpredictability of patients’ behaviors	37	*“There is a wide subjectivity in the evaluation of psychiatric patients: their evaluation and behaviors may change according to the healthcare professional they are referring to in a certain moment”*
Impact of risk-management on the process of care/empowerment/autonomy	36	*“For example, restrictions to outdoor access for some inpatients. They would like to go out at all hours; they would like to have coffee every time they want and they would like to do all the other things they can’t due to the limitations. It is difficult to create a collaborative relationship. How can you tell them ‘You can’t go out’. They don’t understand why.”*
Unsupportive/problematic caregivers	34	*“If a caregiver sees his relative sedated, he may get angry..”*
Potential threats to the therapeutic process/alliance	25	*“Sometimes we avoid involving patients in order to preserve his saneness. In the psychiatric field is difficult to evaluate how much information the patient may tolerate”*
Lack of resources and healthcare/social network	14	*“They often ask us for a cigarette because they get bored. It would be much better to have a walk or a chat with the patient instead of giving him a cigarette. But unfortunately we don’t always have time to do it, even if we are aware it would be the best for the patient.”*
Aggressiveness towards HPs	14	*“If I don’t feel safe as healthcare operator, it is difficult to care for patient safety; I need to first ensure my safety in order to work well.”*
Self-harming patients’ attitudes and behaviours	13	*“It is difficult when they (the patients) do not want to collaborate and it looks like they are doing their best the get things going wrong.”*
Linguistic and cultural barriers	12	*“It is difficult to involve foreign patients, both because of the language and the different culture. They generally stay in contact until they are feeling bad but as soon as they get a bit better, they disappear”*
Being overwhelmed and burnout of HPs	8	*“Verbal aggression is generally underestimated. It may become unpleasant if you (health operator) can’t leave such a feeling at work and you take it at home with you”*
Other	6	*“it is difficult to control the door of the ward and to check if patients have lighters…”*
Total	362	

Building *therapeutic trust/alliance* was considered as the main strategy of HPs facilitating patients’ participation in risk management (18%). Two of the core elements underlying patient empowerment in the clinical process were also identified by HPs as crucial for patient safety: *relying on the experience/knowledge of patients* (14%) and *providing information/educate* (14%).

Additionally, *promoting collaboration with family* (9%), *individualized healthcare plan* (8%), and *relying on the experience/knowledge of* HPs (8%) were also mentioned (see Table 2).

The six most frequent critical issues identified as potential threats to patients’ participation in risk management turned out to be: *inappropriate setting and environment* (e.g., unsafe spaces in hospital psychiatric units, 12%), *patients’ low awareness, reliability* (e.g., ego-syntonic disorders leading to dysfunctional behaviors, believes, and feelings perceived as acceptable by the patient, 12%), followed by aspects referring directly to healthcare providers, such as *poor teamwork* (e.g., poor communication/coordination among the team members, 10%), *HPs inadequately trained or inexperienced* (e.g., HPs not sufficiently skilled to enable patients’ engagement in risk management, 10%), and the *subjectivity of risk-assessment or the unpredictability of patients’ behaviors* (e.g., risk assessment varies according to risk perceptions and risk tolerability, which are partially determined by HPs’ individual characteristics, 10%).

Finally, also the *impact of risk management on the process of care/empowerment/autonomy* was mentioned as critical aspect (e.g., in some cases, pursuing patient safety may lead HPs to take decisions that limit patients’ autonomy and therefore threaten their empowerment, 10%) (see Table 3).

According to participants’ comments, the relevance of each strategy and critical issue varies depending on the context (i.e., thematic area). Thus, the strategies most frequently applied to facilitate patients’ empowerment in the management of therapeutic errors may be inappropriate in other situations, such as dealing with patients’ aggressive behaviour. Additional file 1 shows how specific strategies and critical issues are linked to the thematic areas.

## Discussion

Our study addresses psychiatric patients’ involvement in risk management by investigating the opinions of HPs. Applying a multi-level codification system, we provided a precise overview of the complex role of psychiatric patients in risk management during the diagnostic and therapeutic process.

Our results are represented by the evidence that there is still the necessity to tailor the acquired knowledge about risk management and patient safety as well as the respective protocols, developed in the generic medical setting, to the context of psychiatry, since in this latter setting specific needs and challenges have to be faced. According to HPs contributions, a profound change of perspective is necessary in this adaptive process.

In the following, the key themes/elements as well as the critical issues and strategic implications derived from the focus group discussion have been summarized.

### Patient adherence to risk management activities

In most of the medical fields, patients’ willingness to adhere to safety protocols can be presumed. In psychiatry, this aspect is more controversial, as highlighted also in our study by the high percentage of critical issues discussed in the categories *Therapeutic compliance, Self-harm/suicide,* and *Acute/psychotic symptoms*. Indeed, some psychopathological conditions [20, 22, 32, 33], such as severe depression, may undermine patients’ willingness to take care of their own health and safety and, in the worst cases, even existence (i.e., suicide). Furthermore, psychotic symptoms (e.g., hallucinations) may alter patients’ perceptions of reality and consequently affect the perception of potential sources of risk. Lastly, even personality traits can compromise patients’ compliance to risk management as demonstrated, for instance, by self-harming behaviors occurring in patients with borderline disorder.

Even if the three most frequently cited strategies to promote patients’ involvement turned out to be *Building therapeutic trust/alliance, Providing information/educate,* and *Relying on the experience/knowledge of patients,* HPs considered *Patients’ low awareness, reliability, accessibility* as one of the most significant limitations to the process. These results underline that HPs value patients’ perspective and collaboration considering it as a mean to ensure higher level of safety but, at the same time, feel that this collaboration may be easily questioned and compromised.

This topic has been already widely discussed in the literature [34]. For instance, Tambuyzer and colleagues [35] demonstrated that the process of patient involvement might be affected by both “the person’s *ability* to be involved and his *motivation* or desire to be involved” (p.142). Even if several authors consider patient involvement as a continuum rather than an “all-or nothing” process [36, 37], it seems that HPs still present difficulties in moving along this continuum when they have to decide how and to what extent to involve psychiatric patients with low insight. Thus, this patients’ vulnerability may lead HPs to avoid their involvement instead of trying to tailor the level of empowerment to each patient in each specific phase of the disease.

### Subject at risk

Some of the psychopathological conditions described above determine also another peculiar challenge, which HPs working in psychiatric and emergency departments have to face: the risk of personal aggressions [38, 39]. Therapeutic relationships usually rely on the implicit assumption that patients are in a fragile position and look for HPs’ care and support. Therefore, interpersonal conflicts, even aggressive reactions, may happen but are generally rare and in most of the cases expressed just at a verbal level. In the focus group discussions, critical issues, referring to the category *Aggressive Behavior,* were mentioned twice as much as strategies, suggesting that HPs’ consider this aspect particularly challenging. The element that, according to their suggestion, represents the main strategy in handling patients’ aggressive behavior was hosting patients during the acute phases of their disease, in an in-patient psychiatric ward characterized by a comfortable environment, where their privacy, freedom and safety are protected and guaranteed (see the subcategories of additional file 1: *Appropriate/Inappropriate setting and Environment*). In a recent systematic review on the role of the psychiatric ward as a therapeutic space [40], it has been suggested that psychiatric facilities should be aligned to the current policy on patient-centered healthcare and should therefore be designed following a more holistic approach taking into account structural, organizational, symbolic, and social dimensions. In particular, van der Schaaf et al. [41] found that the availability of private spaces and a high level of comfort were associated with a lower risk of violence.

Nevertheless, it has to be considered that the strategic value attributed by the HPs to the environment might have been determined by the fact that they considered their wards particularly inappropriate. The subcategory *Inappropriate setting and environment* (critical issue) was indeed the most frequently discussed, mentioned also as relevant in referral to other specific categories like *Relapse prevention/symptom management* or *Self-harm/suicide*.

### Objectiveness of risk assessment and Standardizability of patient safety protocols

The subcategory *Subjective risk assessment or unpredictable behaviours* (critical issue) is seen as another peculiar challenge that HPs have to face in dealing with mental illness. Risk assessment of a surgery procedure can be mostly standardized, as it relies on the analysis of objective parameters like cardiac status, type of operation, and level of urgency [42, 43]. Further, the risk of errors in the administration of drugs has been widely reduced in the last few decades with the introduction of some standardized strategies (e.g., double check, guidelines to deal with Look Alike, Sound Alike drugs, the implementation of strategies to reduce interruptions) that have been also mentioned by our participants when commenting the category *Therapeutic error*. Unfortunately, this approach cannot be fully transferred to the psychiatric setting, especially when decision-making affects the risk assessment of patients’ behaviors. For instance, deciding if a patient can leave the in-patient ward for a walk alone or if she/he can receive visits from friends or parents are therapeutic decisions strictly related to the process of care and in some cases also very relevant for patient safety. Obviously, some objective parameters and clinical protocols guide clinicians’ decisions in these circumstances [44] but, considering that part of these measures are based on HPs’ observation, it is implicitly accepted that they are partially affected by the subjective perspective of the observer. Blumenthal and colleagues [45] investigated the relative impact of actuarial and emotive information on mental health professionals’ rating of the risk of violence. In this paper, actuarial risk assessment was based on records, measures, and historical information (e.g., criminal history, family history), while the emotion-based assessment was considered as driven by emotionally laden information (e.g., empathy, insight). The Authors showed that emotive information had a greater impact on risk assessment compared to actuarial information. Thus, these findings call into question the reliability and applicability of safety protocols and guidelines, one of the core tools in safety culture. The issue of subjectivity in risk evaluation has significant implications for the implementation of the so-called “Just Culture” [46–48] in patient safety. This approach aims to find a balance between the strictly non-punitive safety culture, that followed and mitigated the previous tendency to blame individuals involved in medical error, and HPs’ accountability and responsibility [48]. According to our results, “drawing the line between blameless and blameworthy actions”, thus defining HPs’ objective responsibility in adverse events [12], seems to be more problematic and challenging than in other settings.

### Impact of risk management on the process of care and patient empowerment

*“… They would like to go out at all hours; they would like to have coffee every time they want and they would like to do all the other things they can’t, due to the limitations. It is difficult to create a collaborative relationship. How can you tell them: You can’t go out. They don’t understand why.”* Many verbal contributions in the subcategory *Impact of risk-management on the process of care/empowerment/autonomy* (critical issue) similarly addressed HPs’ dilemma in finding the right balance between protecting and empowering the patient. Promoting autonomy and at the same time protecting from risk, is another challenge of the therapeutic relationship in psychiatry, which strongly affects the effectiveness of the intervention. Ethical issues may derive from the necessity to balance patients’ autonomy and safety by limiting patients’ choices. Taking care of mentally ill patients, especially in the acute phases of their disease, implies taking decisions that interfere and limit their freedom and autonomy, contravening the core principle of empowerment, as also admitted by HPs during the focus groups (categories *Compulsory Treatment* and *Physical/Chemical restraints*).

This balancing act is acknowledged by the theory of *Protective Empowerment* [21], which states that the therapeutic relationship, relying on HP’s sensitivity and openness to the interplay between protective and empowering actions, is the basis for fulfilling the simultaneous responsibility for ensuring patient safety and autonomy. Protection is therefore defined as a set of actions, such as reassurance, valuing patients’ needs and expectations or providing information aimed to help the subject to better meet her/his individual needs, including the need to be protected from risk.

**Fig. 2:**
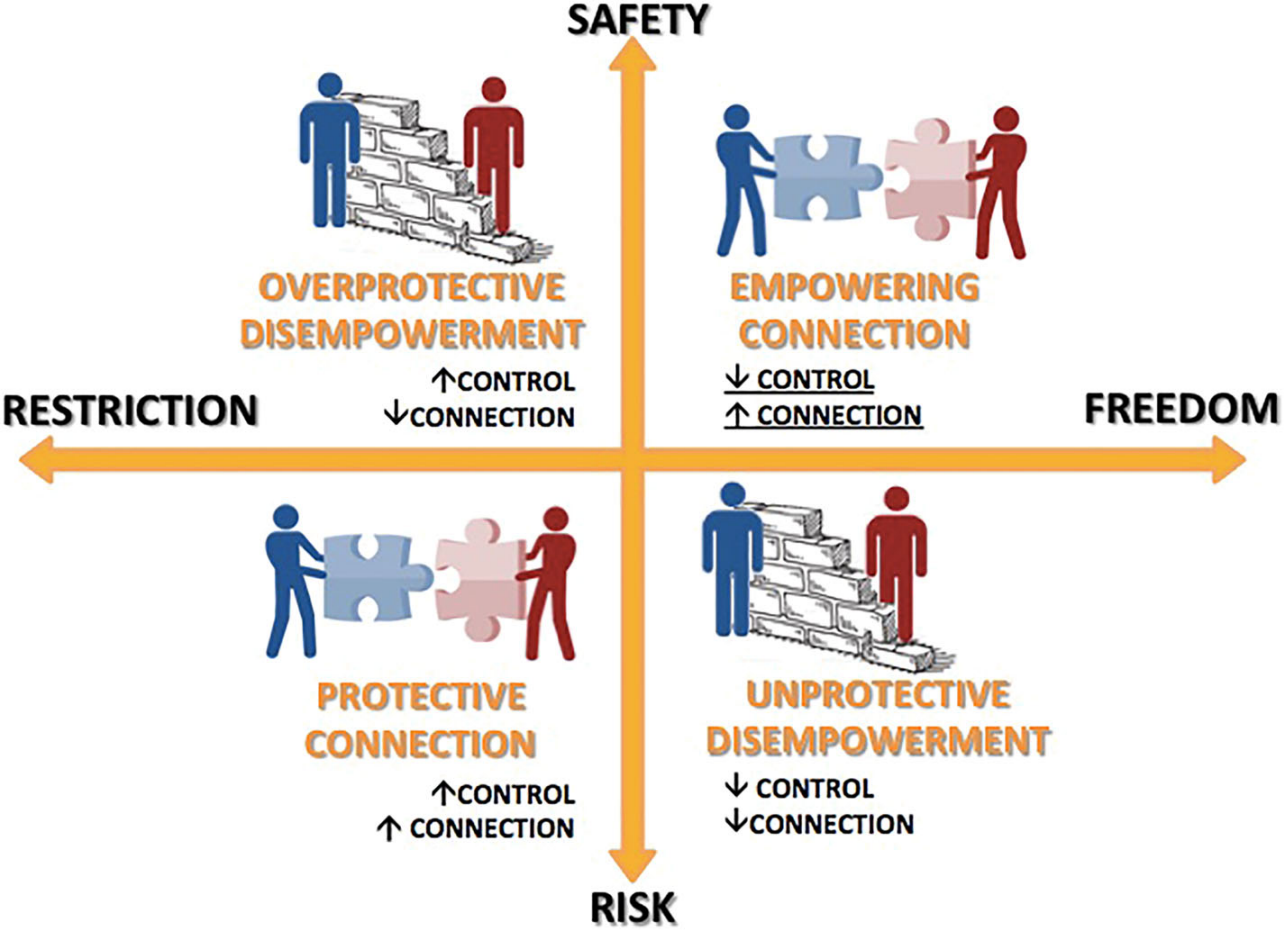
Healthcare providers’ control and emotional connection as mediators of patients’ empowerment in risk management

Following this theory [21] and based on our results, therapeutic interventions in risk management can be defined on two axes (see Fig. 2): Restriction vs. Freedom and Safety vs. Risk.

A “defensive” approach where HPs’ priority is to control and reduce all sources of potential risk for patients - and consequently for themselves (e.g., adverse events, lawsuit) - may lead to the development of an overprotective attitude towards patients (*Overprotective Disempowerment*). In this approach, active involvement and personal freedom are sacrificable, independently from the real level of risk patients might be exposed to. As shown on the opposite quadrant of Fig. 2, excessive freedom in conditions of high risk leads to a misinterpretation of the concept of empowerment and exposes patients to potentially harmful or dangerous conditions (*Unprotective Disempowerment*). What determines the inappropriateness of HPs’ interventions in these two attitudes is, as indicated in the figure, a low or absent connection with the patient. This is another meaningful result that emerged from the analysis of our focus groups: the connection, which refers to the doctor-patient relationship, is the principal assessment and therapeutic tool of clinicians. *Therapeutic compliance* is the category with the highest number of comments and *Building a therapeutic trust/alliance* is, in the subcategory *strategies*, the one most frequently discussed. Connecting with patients provides the clinicians a better estimation of the emotional and cognitive internal world of the patient and consequently reduces the subjectivity in the assessment of risk. An *Empowering connection* and a *Protective connection* are the expression of a real encounter between two persons. Thus, even though they determine very different set of actions, both can be considered appropriate according to the given context.

The other strategies, following *Building therapeutic trust/alliance* in terms of frequency, similarly highlight the importance of tuning with patients, by acknowledging their contribution (*Relying on the experience knowledge of patients),* promoting their medical literacy (*providing information/educate),* and the centrality of their personal world *(promoting collaboration with family and individualized healthcare plane).* Most of these strategies are indicated by the above-mentioned theory of *Protective Empowerment* [21] as key elements in the process of balancing patient safety with patients’ freedom of choice in mental health.

### Limitations

Despite our promising results, there are some limitations that need to be considered in the interpretation of our findings.

First of all, our convenience sample was recruited in the same Mental Health Department. Even if the organization of in- and out-patient services is representative of the Italian services, some organizational and contextual elements, related to the Department, may have affected the discussed topics and therefore limited the generalizability of the results. Moreover, the sample was mainly composed of nurses. Therefore, the discussions during the focus groups may have been affected by their specific perspective. For instance, the thematic area *Diagnostic and Assessment* was poorly discussed, probably because nurses are not primarily involved in these tasks. Although a higher number of doctors in the focus groups may have increased the generalizability of our results and probably deepened the exploration of certain areas, investigating the perspective of the healthcare providers (i.e, nurses) who usually spend a lot of time with patients, has, in our opinion, fostered the robustness of our findings.

The high number of facilitators’ interventions might represent another potential limitation, which may lead to questioning their neutrality. However, from the text analysis of their contributions, many of their interventions aimed to refocus participants to the topics of the discussion. Indeed, in several cases, HPs tended to shift from the concept of patients’ involvement in their own safety to more general considerations about patients’ empowerment in the process of care. This was probably due to the fact that, as above stated, in psychiatry, these two aspects are strongly related and therefore jointly represented in the opinions of participants.

It has also to be taken into account that our results might be seen as unspecific and simply intuitive but up to now, to the best of our knowledge, no previous studies addressed psychiatric patient empowerment in their own safety. Therefore, our results represent a primary summary of the current evidence, which could then be used as background for future quantitative projects assessing, for instance, the extent of psychiatric patients’ involvement in risk management.

Further, we have to consider the impact of translating our findings from Italian to English. Thus, to reduce the potential loss of meaning and to avoid limitations in the analysis of data, we stayed in Italian (i.e., the original language) as long as possible, as suggested by Van Nes et al. [49]. Since all participants, facilitators, and researchers spoke Italian, no language differences hindered the collection of data, the transcription of the focus group discussions, and the development of the coding system. Only in the last phase, preparing the manuscript, one of the authors, fluent in both languages, translated participants’ quotes and the labels of the coding system from Italian to English.

### Implications for research, policy, and clinical practice

The results of our study have several implications for the quality of mental health services and for the direction of future research in risk management.

The most meaningful implication for direct clinical practice and policy makers is the acknowledgement of the key role played by single HPs in the process of risk assessment, adverse event prevention and, more generally, in promoting patient safety in the empowering relationship with the patient. Safety protocols and guidelines can be correctly applied in a therapeutic relationship only if HPs are sufficiently supported in their daily practice. To reach this goal, several actions have been suggested by the participants of the study, such as improving the quality of mental health services’ environment or increasing HPs’ training and supervision. Regarding this last aspect, our results may be considered as reference point in the implementation of educational programs aiming to 1) raise HPs’ awareness of the synergic but also potentially controversial interconnection between empowerment and safety in mental health, and 2) increase the application of patient-centered skills enabling the development of a protective/empowering connection with patients during the process of care and risk management.

Finally, considering that patients’ empowerment is an action requiring the joint collaboration of the two involved parties, patients and HPs, our results have also implications for future research. In particular, the application of our coding system in studies in which psychiatric patients will be engaged in focus groups discussions on this topic, will permit to evaluate if the strategies and critical issues outlined by HPs are similarly perceived by patients.

## Conclusion

In conclusion, safety, although recognized as a crucial element, should not undermine other values or become the singular purpose of psychiatric care. *Empowering* and *protective connection* are the two dimensions in which the process of care and risk management find a mutually beneficial integration. Our results confirm that not just healthcare providers but also patients are supposed to play a key role in enhancing safety. Further studies, actively involving psychiatric patients and even their caregivers as participants, are necessary to fully address the needs of this vulnerable category of patients.

## Additional file

Additional file 1: Sub-categories (strategies and critical issues) divided by thematic area and category. (DOCX 24 kb)

## Data Availability

The datasets used and analyzed during the current study are available from the corresponding author on reasonable request.

## Abbreviations

HP: Healthcare provider
MHD: Mental health department
WHO: World Health Organization
