# Supplementary material for: Patient empowerment in risk management: a mixed-method study to explore mental health professionals’ perspective
Source: BMC Health Serv Res. 2019 Jun 13;19:382. doi: 10.1186/s12913-019-4215-x (PMC6567542; doi:10.1186/s12913-019-4215-x)
Supplement: Supplementary file 1 — Sub-categories (strategies and critical issues) divided by thematic area and category. (DOCX 24 kb) [file 12913_2019_4215_MOESM1_ESM.docx]

**Additional file 1. Sub-categories (strategies and critical issues) divided by thematic area and category.**

| **Thematic area°**  **(level 2)** | **Category°**  **(level 3)** | **Strategy** | **Critical issue** |
| --- | --- | --- | --- |
|  |  | **Sub-categories (n)**  **(Level 4)** | **Sub-categories (n)**  **(Level 4)** |
| Treatment and cure  *Every comment referring to patient’s path of care, either regarding pharmacological treatment or every other therapeutic approach planned for the patient* | Therapeutic compliance (256)  *Every comment referring to psychiatric patient’s compliance to his/her therapeutic program, in terms of pharmacological therapy and of any other aspect regarding the management of disability* | - **Building therapeutic trust/alliance (34)*** - Collecting information (6) - Handling HPs’ emotions (2) - Handling patients’ emotions (1) - Individualized healthcare plan (8) - Motivating the patient (11) - Promoting collaboration with family (7) - Promoting collaboration with other services of the healthcare network (1) - **Providing information/educate (26)** - Relying on the experience/knowledge of HPs (9) - **Relying on the experience/knowledge of patients (24)** - Team-building (8) - Training HPs (5) | - Inappropriate setting and environment (9) - **Patients’ low awareness, reliability, accessibility (22)** - **Poor teamwork (14)** - HPs inadequately trained or inexperienced (8) - Subjective risk-assessment or unpredictable patients’ behaviors (13) - Impact of risk-management on the process of care/empowerment/autonomy (10) - **Unsupportive/problematic caregivers (14)** - Potential threats to the therapeutic process/alliance (13) - Lack of resources and healthcare/social network (2) - Self-harming patients’ attitudes and behaviors (6) - Linguistic and cultural barriers (1) - Overwhelming and burnout of HPs (2) |
|  | Therapeutic error (78)  *Every comment referring to errors in the administration and management of the pharmacological therapy (also known as skill-based or execution errors)* | - Building therapeutic trust/alliance (2) - Collecting information (3) - Individualized healthcare plan (1) - Promoting collaboration with family (2) - **Providing information/educate (12)** - **Relying on the experience/knowledge of HPs (5)** - **Relying on the experience/knowledge of patients (22)** - Team-building (3) | - Inappropriate setting and environment (1) - **Patients’ low awareness, reliability, accessibility (4)** - **Poor teamwork (8)** - **HPs inadequately trained or inexperienced (6)** - Subjective risk-assessment or unpredictable patients’ behaviors (2) - Impact of risk-management on the process of care/empowerment/autonomy (3) - **Potential threats to the therapeutic process/alliance (4)** |
|  | Relapse prevention/symptom management (195)  *Every comment referring to the management of patient’s chronic symptoms and/or of symptoms’ relapse prevention.*  *The symptoms of acute psychotic states falling into the macro-area "emergency management” are not included.* | - Appropriate setting and environment (10) - **Building therapeutic trust/alliance (24)** - Collecting information (3) - Handling HPs’ emotions (3) - Handling patients’ emotions (4) - **Individualized healthcare plan (17)** - Motivating the patient (4) - **Promoting collaboration with family (20)** - Providing information/educate (10) - Relying on the experience/knowledge of HPs (6) - Relying on the experience/knowledge of patients (7) - Team-building (5) - Training HPs (1) | - **Inappropriate setting and environment (13)** - Patients’ low awareness, reliability, accessibility (8) - Poor teamwork (8) - **HPs inadequately trained or inexperienced (12)** - **Subjective risk-assessment or unpredictable patients’ behaviors (12)** - Impact of risk-management on the process of care/empowerment/autonomy (3) - Unsupportive/problematic caregivers (10) - Potential threats to the therapeutic process/alliance (4) - Lack of resources and healthcare/social network (4) - Linguistic and cultural barriers (3) - Being overwhelmed and burnout of HPs (4) |
| Diagnosis and assessment  *Every comment referring to the first diagnosis/assessment of the psychiatric patient* | Onset (6)  *Every comment referring to the first diagnosis at the onset of the symptoms.*  *Comments regarding acute psychotic symptoms fall into this area if the main theme is the diagnosis, while they fall into the macro-area "emergency management" and into the micro-area "acute psychotic status" if the main theme is the management of the acute phase.*  *Comments regarding the management of "chronic" symptoms (i.e., symptoms that remain or reappear after the "first diagnosis") fall into the macro-area "treatment and cure" and into the micro-area "relapse prevention/symptom management".* |  | - **Patients’ low awareness, reliability, accessibility (1)** - **Unsupportive/problematic caregivers (4)** - **Potential threats to the therapeutic process/alliance (1)** |
|  | Stigma (7)  *Every comment referring to stigma usually linked to the first diagnosis and which can lead to "risky" experiences of non-acceptance/denial of the problem that must be appropriately managed to avoid consequences for patient safety* | - **Individualized healthcare plan (2)** - **Promoting collaboration with family (1)** | - **HPs inadequately trained or inexperienced (1)** - **Impact of risk-management on the process of care/empowerment/autonomy (1)** - **Lack of resources and healthcare/social network (2)** |
|  | Diagnostic error (7)  *Every comment referring to diagnostic errors or delays (also known as knowledge-based mistakes or errors based on incorrect reasoning)* | - **Collecting information (1)** - **Promoting collaboration with other services of the healthcare network (2)** - **Providing information/educate (2)** | - **Subjective risk-assessment or unpredictable patients’ behaviors (1)** - **Linguistic and cultural barriers (1)** |
|  | Assessment of social needs (37)  *Every comment referring to the multi-dimensional evaluation of the psychiatric patient* | - Building therapeutic trust/alliance (1) - **Collecting information (2)** - **Individualized healthcare plan (4)** - Motivating the patient (1) - Promoting collaboration with family (1) - Providing information/educate (1) - Relying on the experience/knowledge of HPs (1) - Team-building (1) | - Inappropriate setting and environment (1) - Patients’ low awareness, reliability, accessibility (1) - Poor teamwork (2) - **HPs inadequately trained or inexperienced (3)** - Subjective risk-assessment or unpredictable patients’ behaviors (2) - **Impact of risk-management on the process of care/empowerment/autonomy (5)** - **Unsupportive/problematic caregivers (3)** - Potential threats to the therapeutic process/alliance (1) - **Lack of resources and healthcare/social network (5)** - Being overwhelmed and burnout of HPs (1) |
| Emergency management  *Every comment referring to the diagnosis and assessment of the patient in an acute phase and to the treatment and cure of the patient receiving compulsory medical treatment as well as to the application of restraining and the management of aggression against self and against others* | Self-harm/suicide (36)  *Every comment referring to the management of self-injury or suicidal behavior (including suicidal ideation)* | - **Appropriate setting and environment (5)** - Building therapeutic trust/alliance (2) - Handling patients’ emotions (2) - Promoting collaboration with family (1) - Providing information/educate (2) - **Relying on the experience/knowledge of HPs (5)** - Relying on the experience/knowledge of patients (1) - Team-building (1) | - **Inappropriate setting and environment (6)** - HPs inadequately trained or inexperienced (1) - **Potential threats to the therapeutic process/alliance (2)** - Aggressiveness towards HPs (1) - **Self-harming patients’ attitudes and behaviors (7**) |
|  | Aggressive Behavior (65)  *Every comment referring to aggressive behavior towards others (often considered as “states of psychomotor agitation”)*  *Aggressive behavior towards others associated with substance abuse or organic states are considered as “delirium” and fall into the macro-area “comorbidity”.* | - **Appropriate setting and environment (3)** - **Building therapeutic trust/alliance (4)** - Collecting information (1) - Handling patients’ emotions (1) - Promoting collaboration with family (2) - **Providing information/educate (3)** - Relying on the experience/knowledge of HPs (2) - Relying on the experience/knowledge of patients (2) - **Team-building (3)** - Training HPs (2) | - **Inappropriate setting and environment (10)** - Patients’ low awareness, reliability, accessibility (2) - Poor teamwork (1) - HPs inadequately trained or inexperienced (2) - Subjective risk-assessment or unpredictable patients’ behaviors (3) - Impact of risk-management on the process of care/empowerment/autonomy (1) - Unsupportive/problematic caregivers (2) - **Aggressiveness towards HPs (13)** - **Linguistic and cultural barriers (7)** - Being overwhelmed and burnout of HPs (1) |
|  | Involuntary treatment (21)  *Every comment referring to the management of involuntary treatment, independently of the underlying cause (e.g., psychiatric disease, substance addiction, organic delirium)* | - **Building therapeutic trust/alliance (3)** - Handling patients’ emotions (1) - Individualized healthcare plan (1) - Motivating the patient (1) - **Promoting collaboration with other services of the healthcare network (2)** - Relying on the experience/knowledge of HPs (1) - Team-building (1) | - Inappropriate setting and environment (1) - Patients’ low awareness, reliability, accessibility (1) - Poor teamwork (1) - Subjective risk-assessment or unpredictable patients’ behaviors (1) - **Impact of risk-management on the process of care/empowerment/autonomy (7)** |
|  | Physical/pharmaceutical restraint (12) *Every comment referring to both physical and* *pharmacological restraints, regardless of the underlying cause. The category “compulsory treatment” is used for all comments linked to any treatment “against the will of the patient“ and somehow affecting his/her freedom. The category ”restraint" is used whenever the comments refer to the management of restraints as means with specific drawbacks also in the area of patient safety.* | - Handling patients’ emotions (1) - **Relying on the experience/knowledge of HPs (3)** - Team-building (1) | - Patients’ low awareness, reliability, accessibility (1) - **Impact of risk-management on the process of care/empowerment/autonomy (5)** - Unsupportive/problematic caregivers (1) |
|  | Acute psychotic status (25)  *Every comment referring to the management of acute psychotic states linked to a psychiatric diagnosis. If the acute psychotic state is linked* *to comorbidity (e.g., substance addiction or organic states), then the comment falls into the macro-area “comorbidity”. Aspects linked to the first diagnosis (rather than the management) of an acute psychotic state fall into the macro-area “diagnosis and evaluation” and into the micro-area “onset”.* | - **Building therapeutic trust/alliance (3)** - **Handling patients’ emotions (2)** - Individualized healthcare plan (1) - Promoting collaboration with family (1) - **Providing information/educate (2)** - Relying on the experience/knowledge of HPs (1) - **Relying on the experience/knowledge of patients (2)** - Team-building (1) | - Inappropriate setting and environment (1) - **Patients’ low awareness, reliability, accessibility (2)** - **Poor teamwork (4)** - **Subjective risk-assessment or unpredictable patients’ behaviors (3)** - Impact of risk-management on the process of care/empowerment/autonomy (1) - Lack of resources and healthcare/social network (1) |
| Comorbidity  *Every comment referring to the management of the psychiatric patient who has at the same time a diagnosis of drug abuse or organic comorbidities* | Drug abuse (5)  *Every comment referring to addiction to or abuse of psychoactive substances (including alcohol addiction and abstinence). Comments regarding acute psychotic states directly linked to substance abuse fall into this category. Comments regarding involuntary treatment and use of restraint for patients with substance abuse fall into the macro-area “Emergency management”.* |  | - **Inappropriate setting and environment (3)** - **HPs inadequately trained or inexperienced (2)** |
|  | Organic conditions (4)  *Every comment referring to organic comorbidity (e.g., vascular dementia). Comments regarding acute psychotic states directly linked to comorbidity also fall into this area. Comments regarding involuntary treatment or use of restraint for patients with substance abuse fall into the macro-area “emergency management”.* | - Collecting information (1) | - **Patients’ low awareness, reliability, accessibility (1)** - **HPs inadequately trained or inexperienced (2)** |
| Other  *Every comment not belonging to any of the above-mentioned categories* |  | - Other (3) | - Other (6) |

° Coding definitions for Level 2 and 3 are provided.

* The three most frequent strategies and/or critical issues subcategories at Level 4 are highlighted in bold.
